# The Canadian Partnership Against Cancer Rectal Cancer Project: Protocol for a Pan-Canadian, Multidisciplinary Quality Improvement Initiative to Optimize the Quality of Rectal Cancer Care

**DOI:** 10.2196/15535

**Published:** 2020-01-29

**Authors:** Amandeep Pooni, Selina Schmocker, Carl Brown, Anthony MacLean, Lara Williams, Nancy N Baxter, Marko Simunovic, Alexander Sender Liberman, Sebastien Drolet, Katerina Neumann, Kartik Jhaveri, Richard Kirsch, Erin Diane Kennedy

**Affiliations:** 1 Department of Surgery Mount Sinai Hospital Toronto, ON Canada; 2 Institute of Health Policy, Management, and Evaluation Dalla Lana School of Public Health University of Toronto Toronto, ON Canada; 3 Zane Cohen Centre for Digestive Diseases Mount Sinai Hospital Toronto, ON Canada; 4 Department of Surgery St Paul's Hospital Vancouver, BC Canada; 5 Department of Surgery Cumming School of Medicine University of Calgary Calgary, AB Canada; 6 Department of Surgery The Ottawa Hospital Ottawa, ON Canada; 7 Department of Surgery and Li Ka Shing Knowledge Institute St Michael's Hospital Toronto, ON Canada; 8 Department of Surgery McMaster Universtiy Hamilton, ON Canada; 9 Department of Surgery McGill University Montreal, QC Canada; 10 Department of Surgery Université Laval Quebec City, QC Canada; 11 Department of Surgery Queen Elizabeth II Health Sciences Centre, Victoria General Site Halifax, NS Canada; 12 Joint Department of Medical Imaging University Health Network, Mount Sinai Hospital, Women's College Hospital Toronto, ON Canada; 13 Department of Pathology and Laboratory Medicine Mount Sinai Hospital Toronto, ON Canada

**Keywords:** rectal cancer, quality improvement, knowledge translation

## Abstract

**Background:**

Over the last 2 decades, the use of multimodal strategies, including total mesorectal excision (TME) surgery, preoperative chemotherapy, multidisciplinary case conference, pelvic magnetic resonance imaging, and pathologic assessment using Quirke method, has led to significant improvements in oncologic outcomes for patients with rectal cancer. Although the literature supports claims on the effectiveness of these multimodal strategies, the uptake of these multimodal strategies varies considerably among centers, suggesting that the best evidence is not always implemented into clinical practice.

**Objective:**

This study aims to perform a quality improvement initiative to (1) identify existing gaps in care for these multimodal strategies and (2) implement knowledge translation (KT) interventions to close these gaps to optimize quality of care for patients with rectal cancer across high-volume centers in Canada.

**Methods:**

Process indicators for the selected multimodal strategies to optimize rectal cancer care will be selected and prospectively collected for all patients with stages 1 to 3 rectal cancer undergoing TME surgery. KT interventions, including audit and feedback, opinion leaders, and community of practice, will be implemented to increase the uptake of these clinical strategies.

**Results:**

The uptake of the process indicators over time and the effect of the uptake of the process indicators on short- and long-term oncologic outcomes will be evaluated for each multimodal strategy.

**Conclusions:**

This quality improvement initiative will identify existing gaps in care for the selected multimodal strategies and implement KT interventions to close these gaps. The results of this study will inform further efforts to optimize rectal cancer care.

**International Registered Report Identifier (IRRID):**

DERR1-10.2196/15535

## Introduction

Over the last 2 decades, the increasing use of multimodal strategies has resulted in significant improvements in oncologic outcomes. Although some of this may be attributed to the widespread adoption of total mesorectal excision (TME), multimodal strategies, including the addition of preoperative chemoradiotherapy, have led to further decreases in local recurrence rates and are currently recommended for stage 2 and stage 3 diseases [1-6]. Furthermore, improved pretreatment staging with pelvic magnetic resonance imaging (MRI) has increased the appropriate assignment of preoperative chemoradiotherapy and has assisted with surgical planning by predicting the status of the circumferential resection margin (CRM) status [7,8]. Similarly, pathologic assessments, including the status of the CRM and completeness of the TME, are important quality and prognostic indicators [9,10]. Finally, a multidisciplinary cancer conference (MCC) has been introduced to enhance interdisciplinary communication and coordinate, deliver, and monitor the ideal treatment for each patient [2,6,11].

Although these multimodal strategies have been shown to be effective in the literature, are recommended in published guidelines, and are mandatory components of the Commission on Cancer National Accreditation Program for Rectal Cancer [5,12-14], the implementation of these multimodal strategies varies considerably across centers in North America and Europe [15-20]. This unwarranted variation in the uptake of these multimodal strategies suggests that the best evidence is not always implemented into clinical practice and represents a significant quality gap for patients and providers [21]. To date, there has been little systematic investigation to evaluate the impact of knowledge translation (KT) interventions to reduce variation and optimize the quality of care for patients with rectal cancer.

The Canadian Partnership Against Cancer (CPAC) Rectal Cancer Project is a multiyear, multifaceted quality improvement initiative that will be conducted at 8 high-volume rectal cancer centers across Canada. The objectives of this initiative are to (1) identify existing *evidence to practice* gaps for multimodal care of rectal cancer, including pretreatment MRI, MCC, appropriate use of preoperative radiotherapy, TME surgery, and pathologic assessment using the Quirke method and (2) initiate KT interventions to close these gaps to ensure that all patients with rectal cancer receive optimal and high-quality care.

## Methods

### Prestudy Needs Assessment

Before developing our study protocol, our investigative team conducted a survey to assess the uptake and variation of uptake of multimodal strategies for the treatment of rectal cancer at 8 high-volume centers across Canada. An email survey was sent to a senior surgeon at each of these centers, and they were asked to indicate their center’s current practices for each of the multimodal strategies shown in Table 1. These modalities included routine use of (1) the Quirke method for pathologic assessment, (2) the College of American Pathology (CAP) checklist for a pathologic report, (3) a synoptic operative report, (4) MCC for all patients with rectal cancer, (5) pretreatment MRI for all patients with rectal cancer, (6) specified MRI protocol for rectal cancer, and (7) a synoptic MRI report. The response rate of the survey was 100% (8/8).

The results of this survey showed that there was a significant variation in the uptake of these multimodal strategies across the 8 Canadian centers (Table 1). Most of the hospitals had not formally implemented the Quirke method, and the use of the CAP checklist was variable. Only 2 centers routinely presented patients with a new diagnosis of rectal cancer at MCC, and only 1 center formally documented this MCC decision in the patient chart. Although all centers reported routine use of MRI for local staging of rectal cancer, only 2 centers reported using a standard MRI protocol and synoptic MRI report. No center had implemented all the multimodal strategies, and those that have implemented 1 or 2 of these strategies had seemed to have done this without complete fidelity to optimal practice. On the basis of these survey results, our investigative team felt that it was reasonable to proceed with our quality improvement initiative.

**Table 1:** Prestudy survey assessing uptake of multimodal strategies at 8 high-volume centers.

Multimodal strategy	Hospital							
	1	2	3	4	5	6	7	8
Quirke protocol	N^a^	N	?^b^	N	Y^c^	Y	N	N
College of American Pathology checklist	?	Y	Y	Y	Y	Y	N	Y
Synoptic operative report	Y	Y	N	Y	N	N	Y	Y
Rectal cancer Multidisciplinary Cancer Conference	N	N	Y	N	Y	N	N	N
Routine use of MRI^d^	Y	Y	Y	Y	Y	Y	Y	Y
MRI protocol	N	N	N	N	Y	Y	N	N
MRI synoptic report	N	N	N	N	Y	Y	N	N

^a^N: no.

^b^?: unable to determine.

^c^Y: yes.

^d^MRI: magnetic resonance imaging.

### Study Overview

**Figure 1:**
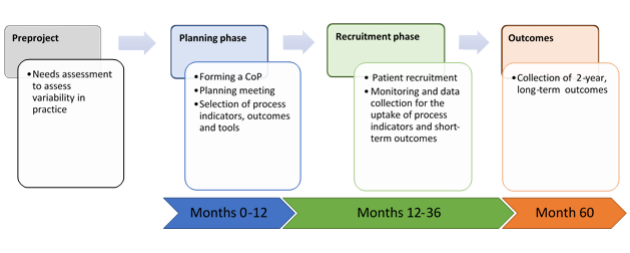
Study overview. CoP: community of practice.

This initiative will be conducted at 8 high-volume rectal cancer centers across Canada and will consist of 2 phases. The first phase is a planning phase that will last 1 year, and the second phase is a recruitment phase that will last 2 years. During this phase, process indicators for the selected multimodal strategies will be collected and monitored, and KT interventions will be conducted to increase the uptake of these process indicators. An overview of the study is provided in Figure 1. Before the start of the study, research ethics approval and data sharing agreements will be obtained.

### Conceptual Framework

**Figure 2:**
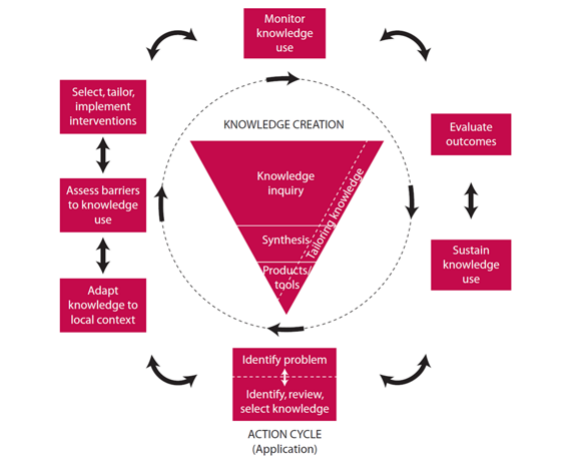
Knowledge to Action cycle.

The conceptual framework for this initiative is the Knowledge to Action (KTA) cycle (Figure 2) [22]. The KTA cycle consists of a knowledge creation funnel and an action cycle. Knowledge creation is conceptualized by an inverted funnel with a vast number of knowledge pieces present in the knowledge inquiry process that are then reduced in number through knowledge synthesis and then an even smaller number of tools or products to facilitate the implementation of the knowledge.

The action cycle is represented by the activities needed for knowledge application. The action cycle is conceptualized as a dynamic process in which all phases in the cycle can influence one another and can also be influenced by the knowledge creation process. The action cycle often starts with an individual or group identifying a problem and the knowledge relevant to solving this problem.

### Knowledge Translation Interventions

KT refers to iterative, multistep processes that involve appraisal of the current quality of care to identify *evidence to practice* gaps, implementation of KT interventions to address these gaps, and outcome measurement to evaluate the effect of these employed interventions [23-26]. For this initiative, the main KT interventions will be a community of practice (CoP), audit and feedback, and opinion leaders.

A CoP is defined as “a group of people who share a concern, a set of problems, or a passion for a topic, and who deepen their knowledge and expertise in an area by interacting on an ongoing basis,” and, more recently, CoPs have emerged as a promising form of KT intervention [27,28]. Through their collaborative and informal structure, it is proposed that CoPs reduce professional and organizational boundaries by facilitating the dissemination of explicit and tacit knowledge among members, reducing professional isolation, and encouraging group innovation [28,29]. The utility of CoPs has been demonstrated in several health care settings, where they have been used to improve compliance with evidence-based guidelines [30].

Audit and feedback is a widely used strategy to improve health care practice and has been shown to be an effective strategy to promote behavior change among physicians [31]. Improvements from audit and feedback have been attributed to the intrinsic effects of friendly competition among participants, self-regulation based on performance goals, and the Hawthorne effect [31]. Specific to rectal cancer care, several European studies have demonstrated improvements in survival and quality of care after implementing national rectal cancer audits, including those without comparative feedback [32,33].

Physician leaders act as role models and opinion leaders to effect behavior change in other physicians. Physician leaders have been shown to increase compliance with implementation guidelines and help to ensure that changes are sustained after implementation is complete [34-36].

### Participating Centers

A total of 8 high-volume rectal cancer centers across Canada will participate in this initiative and represent a convenience sample. At each center, there will be a designated site lead who will be in charge of managing the project locally at their institution.

### Planning Phase (Year 1)

During the planning phase of the study, our investigative team will assemble a pan-Canadian, multidisciplinary CoP and organize an in-person planning workshop with the CoP to finalize the study protocol.

The investigative team will form the CoP by inviting a colorectal surgeon at each of the 8 sites to be the site lead and act as a champion for the study at their institution. Each of these surgeons will be asked to invite a radiologist, radiation oncologist, medical oncologist, and pathologist from their respective centers to be a member of the CoP and site lead at their center for their respective specialty.

The CoP members will be invited to participate in an in-person planning workshop to finalize the study protocol. This will involve a modified Delphi method with the CoP to select process indicators and outcomes to measure the uptake of the multimodal strategies to optimize rectal cancer care, including (1) pretreatment MRI, (2) MCC, (3) appropriate use of radiotherapy, (4) TME, and (5) pathologic assessment using the Quirke method. Before the workshop, a survey will be circulated to the CoP with suggested process indicators and outcomes for each of the multimodal strategies, and the CoP members will be asked to rate each of these process indicators and outcomes on a categorical scale from 1 to 5 based on clinical importance. The CoP members will also be encouraged to provide additional process indicators and outcomes on the survey. At the workshop, the survey results and best available evidence for each of the suggested process indicators and outcomes will be presented. This will be followed by a moderated multidisciplinary discussion and an anonymous vote to include or exclude each of the suggested process indicators and outcomes. All the suggested process indicators and outcomes in which 90% or more of the CoP vote to keep will be used as the final set of process indicators and outcomes for the study. Following the selection of the process indicators and outcomes, the CoP will discuss specific tools that may be used to capture the selected process indicators and outcomes. The goal of this discussion will be to identify specific tools that are already being used successfully at the participating centers and to modify these as necessary for the purpose of the study. Therefore, at the end of the workshop, the study framework with recommended process indicators, outcomes, and tools will have been finalized by the CoP. The suggested process indicators and tools included in the preworkshop survey are shown in Table 2. The suggested short-term oncologic outcomes included in the preworkshop survey are completeness of TME, CRM status, and lymph node retrieval. The suggested long-term oncologic outcomes included in the preworkshop survey are local recurrence, distant metastasis, overall survival, and disease-free survival at 2 years.

The site leads at each participating center will be responsible for presenting the final study protocol to their colleagues at a study launch. At the study launch, the site leads will also obtain feedback from their colleagues regarding facilitators and barriers to implementation at their institution. Although the final process indicators and outcomes will not be modifiable, the site leads will be encouraged to modify the recommended tools to facilitate the implementation of the multimodal strategies at their center locally based on feedback from their colleagues.

**Table 2:** Suggested process indicators and tools.

Multimodal strategy	Suggested process indicators	Suggested tools
All patients with rectal cancer should have a pretreatment MRI^a^	Performance of pretreatment MRI and MRI report includes *T-category, N-category*, *and circumferential margin status*	Synoptic MRI report
All patients with rectal cancer should have their case presented at MCC^b^	Presentation of case at MCC and MCC report generated for all cases	Synoptic MCC report
All patients with rectal cancer should receive high-quality TME^c^ surgery	Completeness of TME documented in operative note and preoperative stoma marking	Synoptic operative report
All rectal cancer specimens should have pathologic assessment using the Quirke method	Use of the Quirke method	Quirke method gross specimen checklist and College of American Pathologist checklist
All patients with locally advanced rectal cancer should receive neoadjuvant CRT^d^	CRT for all patients with stages 2 and 3 rectal cancer and peer review of radiotherapy treatment plan	—^e^

^a^MRI: magnetic resonance imaging.

^b^MCC: multidisciplinary cancer conference.

^c^TME: total mesorectal excision.

^d^CRT: chemoradiotherapy.

^e^No tool identified.

### Recruitment Phase (Years 2 and 3)

The recruitment phase of the study will consist of capturing the selected process indicators and outcomes for all patients with a new diagnosis in a consecutive and prospective manner at each center.

### Patient Sample

Eligible patients will include those with a (1) new diagnosis of stage 1 to 3 biopsy-proven rectal adenocarcinoma; (2) located 15 cm or less from the anal verge; and (3) undergoing curative-intent TME surgery at the participating center. Patients with stage 4 rectal cancer or not requiring TME surgery will be excluded. On the basis of center volumes, it is expected that each center will recruit 50 to 100 rectal cancer patients per year; therefore, data will be collected for 600 to 800 patients with rectal cancer over the recruitment period. Participating physicians will be responsible for identifying potential patients for the study, and at each site, study coordinators will obtain informed consent. Before the start of the recruitment phase, Research Ethics Board approval and data sharing agreements will be obtained from all participating sites.

### Data Collection

A dedicated research coordinator at each site will collect process indicators and short-term oncologic outcomes for each patient, and the research coordinator will abstract these data into a Web-accessible database designed for the study. The hard copies of deidentified patient data, including MRI reports, MCC reports, operative reports, and pathology reports, will also be collected for quality assurance and central review. Moreover, 2 years following the completion of patient recruitment, the long-term oncologic outcomes will be collected for each patient.

Every 3 months, an interim report will be generated for process indicators and short-term outcomes for the entire cohort as well as each individual center. Results will be anonymized for all hospitals outside the home hospital, and a total of 7 reports over the recruitment period will be generated. Each site will be encouraged to identify gaps in care at their center and work together to develop local strategies to close gaps in care. The CoP will participate in a teleconference after each reporting cycle to discuss the reports, evaluate each other’s progress, identify barriers to success, and discuss strategies to overcome these barriers. After the first year of recruitment, a second, in-person meeting is planned. At this meeting, high-performing hospitals will be asked to present their practices and implementation strategy to the other sites, and the sustainability of the multimodal strategies will be discussed.

### Data Analysis

For the final data analysis, each process indicator will be reviewed and presented graphically using standard quality improvement tools including statistical process control and run charge to identify change trends. To account for the lack of independence in the outcome measurements (ie, patients clustered within centers), generalized estimating equations models will be used to evaluate group differences for the indicators over time and between centers. Center-specific evaluation of selected outcomes over time will employ standard statistical analysis, such as logistic regression. Incorporated in all analyses will be an evaluation of the appropriateness of statistical technique and assumptions. Both short- and long-term oncologic outcomes will be reported, and Kaplan-Meier curves for disease-specific and overall 2-year survival will be generated.

The final analysis will evaluate the effect of the uptake of the process indicators on short- and long-term oncologic outcomes.

## Results

The CPAC is funding this study. Data collection and central review are currently ongoing and will be completed by December 2019.

## Discussion

### Principal Findings

The CPAC Rectal Cancer Project is a multiyear, multifaceted KT initiative that aims to identify existing *evidence to practice* gaps in rectal cancer care and to implement KT interventions to close these gaps with the overall goal to optimize the quality of care and outcomes for patients with rectal cancer. The results of this study will be highly relevant, as they will not only provide insight into the current status of rectal cancer care but also evaluate the effectiveness of targeted KT interventions to optimize care and provide data for the development of national benchmarks.

There are several unique features of this proposed study. First, an integrated KT approach is employed in both the planning and recruitment phases of the study through the use of the CoP. It is expected that by involving the CoP or knowledge users early in the planning process of the study, the results will be more relevant and more likely to be used in clinical practice by the CoP members and their colleagues. The CoP is also expected to increase stakeholder *ownership* and *engagement* with the study and increase the likelihood of successful implementation of the multimodal strategies and long-term sustainability. Another important attribute of the CoP is that it is multidisciplinary, and this is expected to foster more meaningful interdisciplinary discussions, collaboration, and sharing of best practice across specialties. In this regard, we plan to have the process indicators and outcomes selected by all the members of the CoP.

Following the completion of the study, our group will have unique patient-level, process indicator, and outcome data, and we will be able to use these data to set national and multidisciplinary benchmarks for optimal rectal cancer care in Canada. We will also be able to recommend successful KT interventions that will provide the opportunity to scale this approach out to other centers. Finally, there will be standardized procedures and protocols at the 8 participating centers, and the CoP will be able to continue to collaborate and conduct future trials with a coordinated infrastructure.

Some of the limitations of this study are as follows. First, all the participating centers are high-volume academic centers and may not be generalizable to other practice settings or be representative of rectal cancer care on a national level. Second, as this study is focused on the uptake of the process indicators, the study may be relatively underpowered to detect significant differences for both short- and long-term oncologic outcomes. Finally, the sustainability of the study and planning for sustainability of the multimodal strategies following completion of the study will be critical.

### Conclusions

The CPAC Rectal Cancer Project is a quality improvement initiative to identify existing *evidence to practice* gaps in rectal cancer care and implement KT interventions to close these gaps to optimize the quality of care for patients with rectal cancer in Canada. The multidisciplinary CoP is a unique feature of this study and is expected to increase the successful implementation and sustainability of the multimodal strategies for optimal rectal cancer care. The results of the study will be highly relevant, as they will show the current uptake of multimodal strategies for optimal rectal cancer care and the effectiveness of targeted KT interventions to improve the uptake of these multimodal strategies.

## Appendix

Multimedia Appendix 1 Peer Review Funding Reports from Canadian Partnership Against Cancer.

## Abbreviations

CAP: College of American Pathology
CoP: community of practice
CPAC: Canadian Partnership Against Cancer
CRM: circumferential resection margin
KT: knowledge translation
KTA: Knowledge to Action
MCC: multidisciplinary cancer conference
MRI: magnetic resonance imaging
TME: total mesorectal excision
