# Supplementary material for: The Canadian Partnership Against Cancer Rectal Cancer Project: Protocol for a Pan-Canadian, Multidisciplinary Quality Improvement Initiative to Optimize the Quality of Rectal Cancer Care
Source: JMIR Res Protoc. 2020 Jan 29;9(1):e15535. doi: 10.2196/15535 (PMC7016615; doi:10.2196/15535)
Supplement: Multimedia Appendix 1 [file resprot_v9i1e15535_app1.pdf]

January 31, 2014

Dr. Erin Kennedy
Mount Sinai Hospital
600 University Ave.
Toronto, Ontario M5G 1X5

Dear Dr. Kennedy:

Re: **Request for Proposals # RP431-2013-01-05 for Accelerated implementation diffusion of quality initiatives for rectal cancer across Canada .**

On behalf of the Canadian Partnership Against Cancer (Partnership), thank you for your submission in response to the above RFP. Following a competitive application process, all proposals were adjudicated by a panel consisting of external experts to ensure alignment with the objectives of the Accelerated Diffusion of Strategic Quality Initiatives for Diagnosis and Treatment of Cancer.

Following this thorough review, we are pleased to inform you that your proposal was selected for funding. Feedback from the review panel addressed certain recommendations which will be an important first step in negotiating the funding agreement. Copies of the evaluation panel reports are attached. The feedback and next steps will be discussed in a meeting to be scheduled in the near future.

Thank you for your support and commitment to advancing Quality Initiatives for Diagnosis and Treatment of Cancer. We look forward to working with you.

Sincerely,

Samoya Lloyd

Samoya Lloyd
Manager, Procurement
Canadian Partnership Against Cancer

CANADIAN PARTNERSHIP AGAINST CANCER | PARTENARIAT CANADIEN CONTRE LE CANCER

Request For Proposal (RFP) No. RP431-2013-01:
Accelerated Diffusion of Strategic Quality Initiatives for Diagnosis and Treatment of Cancer

Scientific Officer Report For Proposal:
Accelerated Implementation Diffusion of Quality Initiates for Rectal Cancer Across Canada

Lead Applicant:
Erin Kennedy

Overall Summary of Proposal:
This project proposes to accelerate implementation of established quality initiatives for rectal cancer including Total Mesorectal Excision (TME) surgery and assessment, multidisciplinary cancer conferences, and Magnetic Resonance Imaging (MRI) using a multi faceted knowledge translation (KT) strategy. While all have proven to improve clinical outcomes, there is significant variation in uptake and use of these quality initiatives across the country, with no centre having implemented all three initiatives. This project will involve 8 high volume centres across Canada and will include surgeons, radiologists, radiation oncologists, medical oncologists and pathologists involved in the care of rectal cancer patients as well as patient and family advisors.
The Applicant has suggested that successful implementation of these quality initiatives will (i) lead to improved rectal cancer care and clinical outcomes for rectal cancer patients and (ii) set a national standard for these quality initiatives across Canada.

Strengths:
This is a very important and relevant issue with supporting evidence on impact to clinical outcomes. This Proposal is multi-jurisdictional with 8 sites in 6 provinces, involving a minimum of 1000 patients from across the country. The team has done a great job identifying barriers and facilitators so that KT interventions can be tailored to the needs of each site. The Reviewers expressed confidence in their sustainability plan since each site has committed funds to maintain the database and further disseminate within their own jurisdictions beyond the funding period. The patient engagement plan was robust and process outcomes appeared to be measurable and realistic.

Weaknesses:
In the absence of data, there were concerns about the feasibility of implementing all 3 initiatives in each centre concurrently. Particularly, given the variability in baseline practice between centres, mapping out the same timeline and implementation plan for each centre appeared unrealistic. Some of the goals also appeared potentially unrealistic such as the multidisciplinary

cancer conference for all newly diagnosed patients, as well as the emphasis on the use of MRI. Further clarification surrounding the use of the Tailoring Grid provided in Figure 2 would have been helpful to clearly understand the process.

The team composition and process for selecting site leaders could have been further developed. For example, the team did not include medical oncology who play an integral role in the management of these patients. The role of project coordinators in each centre was not clearly articulated. There was also no site lead identified for British Columbia, and the proposed self-selection of site leaders did not seem optimal compared to matching underperforming sites to a site leader and jurisdiction that is doing well with a particular initiative.

Budget:

The budget was lacking detail and not well justified. For example, a large portion of the budget was dedicated to salaries for project coordinators whose roles and responsibilities were unclear. There was no budget built in for site visits for underperforming sites as outlined in the risk management plan. While the in-kind contribution was extensive and demonstrated commitment, there was concern that the proposed work may be too ambitious given the proposed budget.

CANADIAN PARTNERSHIP AGAINST CANCER | PARTENARIAT CANADIEN CONTRE LE CANCER

PROPOSAL NUMBER: RP431-2013-01-05

PRIMARY REVIEWER REPORT

Reviewer Name

Title of Proposal
Accelerated Implementation Diffusion of Quality Initiatives for Rectal Cancer Across Canada

Primary Applicant(s)
Erin Kennedy

Funding Amount Requested
$1,000,000

Overall Summary of Proposal:

- Overview: This project proposes to accelerate implementation of established quality initiatives for rectal cancer including TME surgery and assessment, multidisciplinary cancer conference and MRI using a multi faceted knowledge translation strategy. While all have proven to improve clinical outcomes, there is significant variation in uptake and use of these quality initiatives across the country, with no centre having implemented all three. This project will involve 8 high volume centres across Canada and include surgeons, radiologists, radiation oncologists, medical oncologists and pathologists involved in the care of rectal cancer patients as well as patient and family advisors.
- Methodology: The plan is to have Site Leads attend a national workshop to achieve consensus on selected patient and process level outcomes for each of the quality initiatives including target bench marks, standardized protocols and processes and KT interventions. Patients will also attend this workshop and will be actively engaged in the website development. The Site Leads will then present the overall study plan to their colleagues at a Project Launch meeting and assess barrier and facilitators to implementation of the quality initiatives at their centre. Following completion of the KT interventions including 3 pan-Canadian webinars and list-serv, patient recruitment and data collection for audit and feedback will commence. A report with the results for each of the selected patient and process level outcomes will be issued to each centre every 3 months for a total of 7 cycles (21 months).
- Outcomes: Each of the selected patient level and process level outcomes, including rate of positive CRM will be evaluated (i) relative to the target bench marks set at the start of the study and (ii) the change in these selected outcomes over time at the individual hospital level and national level.
- Relevance: This project is highly relevant as successful implementation of these quality initiatives will (i) lead to improved rectal cancer care and clinical outcomes for rectal cancer patients and (ii) set a national standard for these quality initiatives across Canada.

PROPOSAL NUMBER: RP431-2013-01-05

Overall Assessment of Major Strengths and Weaknesses of Proposal:
Review Criteria is listed in *Appendix III* of the *Review Process & Reviewer Instructions* guide

Major strengths:
- Strong evidence to support that quality of rectal cancer care has an impact on clinical outcomes; proposed initiatives have all been shown to improve clinical outcomes.
- Multijurisdictional – 8 sites, 6 provinces involving minimum of 1000 patients from across the country.
- Use of knowledge translation strategies that have been used effectively in other settings.
- Use of effective strategies for effecting behavior change (opinion leaders & audits/feedback).
- Use of tailoring grids to identify barriers and facilitators, so KT interventions can be tailored to needs of each site Project team with varying skills and background, with experience in managing grants.
- Each site has committed to finding funding to sustain data base post project (resources are small, so high likelihood of sustainability).
- Patients actively engaged, with a plan for consultant with expertise in Patient Engagement to work with patients and the team to ensure patients are used appropriately as health care advisors.
- Patient and process outcomes selected to evaluate quality initiative are measurable, realistic and good indices.

Major weaknesses:
- No site has implemented all 3 initiatives, and there is wide variation across the 8 sites.
- No pilot has been conducted nor is there a plan to do one as part of this project.
- Timelines and plans for implementation are all the same, but extent of change in practice is variable, and in some centres very significant.
- A more multijurisdictional project team is preferred.
- BC has not identified its Site Leaders other than the Surgical Lead who is on the Project Team.
- Plan for underperforming sites include self selection of site leader rather than matching an underperforming site to a site leader and jurisdiction that is doing well in that domain.

Comments on Budget:

- The budget is detailed, with justification for budget well supported by project plan. The budget reflects project milestones and deliverables. The in kind contributions of each team member are outlined. While the project plan does speak to the potential need for additional site visits for underperforming areas, the budget does not seem to account for this contingency.

Specific Issues to be flagged (if applicable):

- Wide variation in starting points for each jurisdiction.

PROPOSAL NUMBER: RP431-2013-01-05

Additional Comments to Applicants (if applicable):
- Ambitious project that will have to be managed carefully in order for the project to be successful.

CANADIAN PARTNERSHIP AGAINST CANCER | PARTENARIAT CANADIEN CONTRE LE CANCER

PROPOSAL NUMBER: RP431-2013-01-05

SECONDARY REVIEWER REPORT

Reviewer Name

Title of Proposal

Accelerated Implementation Diffusion of Quality Initiatives for Rectal Cancer Across Canada

Primary Applicant(s)

Erin Kennedy

Funding Amount Requested

$1,000,000

Please note: **Text boxes do not expand**

Overall Assessment of Major Strengths and Weaknesses of Proposal:
Review Criteria is listed in *Appendix III* of the *Review Process & Reviewer Instructions* guide

- In this proposal, the authors contend that while there is strong evidence to support that the quality of rectal cancer care has a major impact on local recurrence, survival and quality of life, to date quality initiatives for rectal cancer care have not been standardized and have been relatively ad hoc. This had led to incomplete uptake of these quality initiatives and evaluation of their effectiveness. Therefore, this proposed study will aim to accelerate implementation diffusion of established, quality initiatives for rectal cancer including TME surgery and pathology assessment, multidisciplinary cancer conference (MCC) and MRI using a multi-faceted knowledge translation (KT) strategy. This is not as novel as there have been other similar initiatives in this area in other countries.
- This project has a strong team; however, I note that there are no Medical Oncology leads even though rectal cancer requires multidisciplinary input. Having said that, each participating centre will have a Site Lead from Surgery, Radiology, Pathology, and Radiation Oncology responsible for championing and implementing the quality initiatives locally at their centre.
- The investigators propose a good implementation diffusion plan. Specifically, they will use the Knowledge to Action Cycle (KTA) that includes an inner funnel for the production of knowledge tools and an outer cycle outlining steps where potential knowledge translation interventions are utilized. The implementation diffusion plan for the project will include a multi-faceted knowledge translation strategy informed by the KTA outer cycle including: identification of a quality gap in care and selection of knowledge products to close the identified gap; adaptation of selected knowledge products; assessments of barriers to knowledge uptake; selection, tailoring and implementation of interventions; monitoring knowledge uptake; and evaluation of outcomes and efforts to sustain knowledge use.

PROPOSAL NUMBER: RP431-2013-01-05

Comments on Budget:

- Budget is extremely high for proposed work. Do we need coordinators at EACH of the sites? Can tasks be completed online?

Specific Issues to be flagged (if applicable):

Additional Comments to Applicants (if applicable):
